# Construction and validation of a novel tumor morphology immune inflammatory nutritional score (TIIN score) for intrahepatic cholangiocarcinoma: a multicenter study

**DOI:** 10.1186/s12885-024-12375-7

**Published:** 2024-05-23

**Authors:** Haofeng Zhang, Guan Huang, Qingshan Li, Yanbo Wang, Zhenwei Yang, Pengyu Chen, Hao Yuan, Kunlun Chen, Bo Meng, Haibo Yu

**Affiliations:** 1 Department of Hepatobiliary and Pancreatic Surgery, People’s Hospital of Zhengzhou University, Zhengzhou, 450000 China; 2 Department of Hepatobiliary and Pancreatic Surgery, Henan Provincial People’s Hospital, Zhengzhou, 450000 China; 3 Department of Hepatobiliary and Pancreatic Surgery, The First Affiliated Hospital of Zhengzhou University, Zhengzhou, 450000 China; 4 Department of Hepatobiliary and Pancreatic Surgery, Cancer Hospital of Zhengzhou University, Zhengzhou, 450000 China

**Keywords:** Intrahepatic cholangiocarcinoma, Tumor morphology, Immunity, Inflammation, Nutrition, Nomogram, Prognosis

## Abstract

**Background:**

Tumor morphology, immune function, inflammatory levels, and nutritional status play critical roles in the progression of intrahepatic cholangiocarcinoma (ICC). This multicenter study aimed to investigate the association between markers related to tumor morphology, immune function, inflammatory levels, and nutritional status with the prognosis of ICC patients. Additionally, a novel tumor morphology immune inflammatory nutritional score (TIIN score), integrating these factors was constructed.

**Methods:**

A retrospective analysis was performed on 418 patients who underwent radical surgical resection and had postoperative pathological confirmation of ICC between January 2016 and January 2020 at three medical centers. The cohort was divided into a training set (*n* = 272) and a validation set (*n* = 146). The prognostic significance of 16 relevant markers was assessed, and the TIIN score was derived using LASSO regression. Subsequently, the TIIN-nomogram models for OS and RFS were developed based on the TIIN score and the results of multivariate analysis. The predictive performance of the TIIN-nomogram models was evaluated using ROC survival curves, calibration curves, and clinical decision curve analysis (DCA).

**Results:**

The TIIN score, derived from albumin-to-alkaline phosphatase ratio (AAPR), albumin–globulin ratio (AGR), monocyte-to-lymphocyte ratio (MLR), and tumor burden score (TBS), effectively categorized patients into high-risk and low-risk groups using the optimal cutoff value. Compared to individual metrics, the TIIN score demonstrated superior predictive value for both OS and RFS. Furthermore, the TIIN score exhibited strong associations with clinical indicators including obstructive jaundice, CEA, CA19-9, Child–pugh grade, perineural invasion, and 8th edition AJCC N stage. Univariate and multivariate analysis confirmed the TIIN score as an independent risk factor for postoperative OS and RFS in ICC patients (*p* < 0.05). Notably, the TIIN-nomogram models for OS and RFS, constructed based on the multivariate analysis and incorporating the TIIN score, demonstrated excellent predictive ability for postoperative survival in ICC patients.

**Conclusion:**

The development and validation of the TIIN score, a comprehensive composite index incorporating tumor morphology, immune function, inflammatory level, and nutritional status, significantly contribute to the prognostic assessment of ICC patients. Furthermore, the successful application of the TIIN-nomogram prediction model underscores its potential as a valuable tool in guiding individualized treatment strategies for ICC patients. These findings emphasize the importance of personalized approaches in improving the clinical management and outcomes of ICC.

**Supplementary Information:**

The online version contains supplementary material available at 10.1186/s12885-024-12375-7.

## Introduction

Intrahepatic cholangiocarcinoma (ICC) is the second most prevalent malignant tumor of the liver, occurring after hepatocellular carcinoma (HCC), and originating from the intrahepatic cholangiocytes [1, 2]. It constitutes approximately 10% to 20% of primary liver malignancies [2, 3]. The incidence of ICC has exhibited a substantial global increase of approximately 140% over the past few decades [4]. Although adjuvant therapeutic modalities such as chemotherapy, immunotherapy, and targeted therapy are gradually emerging, radical surgical resection remains the established standard for the treatment of ICC [5, 6]. Nevertheless, due to its insidious onset and high recurrence rate, the five-year postoperative survival rate for ICC is merely 10–20% [7]. Thus, it is crucial to develop a dependable preoperative scoring system that can determine the potential benefit of surgery and subsequently facilitate the formulation of personalized treatment plans.

Growing evidence indicates the significant role of tumor morphology, immune function, inflammatory levels, and nutritional status in the development and progression of ICC [8–11]. Various indicators derived from preoperative blood tests and imaging have demonstrated a strong association with prognosis in different cancers, including ICC [12–16]. For instance, elevated tumor burden score (TBS) and systemic immune inflammation index (SII) are closely linked to unfavorable postoperative survival outcomes [8, 17–19], while higher levels of prognostic nutritional index (PNI) generally indicate a favorable prognostic status [20–22]. However, a single index fails to capture the entirety of a patient’s tumor morphology, immune function, inflammatory levels, and nutritional status. Consequently, our study aimed to assess multiple identified indicators and scores, culminating in the development of a novel score, the TIIN score, which integrates tumor morphology, immune function, inflammatory levels, and nutritional status. Additionally, we constructed and evaluated a prediction model based on this score.

## Methods

### Patient selection

This study included patients who underwent radical surgical resection for ICC between January 2016 and January 2020 at three medical institutions: People’s Hospital of Zhengzhou University, Cancer Hospital of Zhengzhou University, and First Affiliated Hospital of Zhengzhou University. For the definition of radical surgical resection: complete removal of all tumor lesions identifiable preoperatively and intraoperatively, with histopathological examination confirming negative margins (margin width ≥ 10 mm); if there is direct invasion of organs or tissues by the tumor, confirmation of negative margins through histopathological examination is also required after combined resection; absence of extrahepatic distant metastasis and invasion of major blood vessels. Additionally, the scope of lymph node dissection is as follows: based on the tumor location, for lesions originating from the left lobe of the liver, the lymph node dissection range includes the hepatoduodenal ligament, lesser omentum to the gastric lesser curvature, and lymph nodes near the cardia; for lesions originating from the right lobe of the liver, the lymph node dissection range includes the hepatoduodenal ligament, portal fissure, and lymph nodes behind the pancreas.

Inclusion criteria consisted of: 1) confirmed ICC diagnosis by postoperative pathology; 2) age ≥ 18 years; 3) no preoperative anticancer treatment; 4) absence of concurrent malignancies. Exclusion criteria encompassed: 1) perioperative deaths; 2) hematologic and autoimmune diseases; 3) incomplete clinical or laboratory data; 4) tumor recurrence necessitating secondary surgery; 5) incomplete follow-up data. A total of 418 patients met the aforementioned criteria and were included in the study. Among them, 272 patients from People’s Hospital of Zhengzhou University and Cancer Hospital of Zhengzhou University constituted the training set, while 146 patients from First Affiliated Hospital of Zhengzhou University formed the validation set. All enrolled patients were assessed using the 8th edition of the American Joint Committee on Cancer (AJCC) staging system and were followed up until January 2023.

This study obtained ethical approval from the Ethics Committee of Zhengzhou University People’s Hospital (No. XHEC-JDYXY-2018–002) and other participating centers prior to initiation. Informed consent was obtained from all patients prior to the commencement of the study.

### Clinical variables

The collected clinical and pathological data of the patients encompassed the following variables: gender, age, obstructive jaundice, hepatitis B virus (HBV) infection, Child–pugh grade, tumor differentiation, perineural invasion, microvascular invasion, and the 8th edition of the American Joint Committee on Cancer (AJCC)-TNM classification. Additionally, laboratory tests were conducted one week prior to surgery, including measurements of alpha fetoprotein (AFP), carcinoembryonic antigen (CEA), carbohydrate antigen199 (CA19-9), alanine transaminase (ALT), aspartate transaminase (AST), alkaline phosphatase (ALP), gamma-glutamyltransferase (GGT), albumin, globulin, bilirubin, prothrombin time (PT), fibrinogen (FIB), white blood cell count (WBC), hemoglobin (HGB), lymphocyte count (LY), neutrophil count (NE), monocyte count (MO), and platelet count (PLT). Furthermore, preoperative imaging was utilized to determine the tumor count and diameter of the patients.

Sixteen indicators related to tumor morphology, immune function, inflammatory levels, and nutritional status were derived from the aforementioned variables. These indicators include albumin–bilirubin (ALBI), ALT, AST, albumin-alkaline phosphatase ratio (AAPR), albumin–globulin ratio (AGR), FIB, gamma-glutamyl transpeptidase-to-platelet ratio (GPR), hemoglobin-albumin-lymphocytes-platelets (HALP), monocyte-to-lymphocyte ratio (MLR), neutrophil-to-lymphocyte ratio (NLR), platelet-to-lymphocyte ratio (PLR), PNI, PT, SII, TBS, and total tumor volume (TTV). The calculation formulas for these indicators are as follows: ALBI: log_10_(bilirubin [mol/L]) × 0.66—albumin [g/L] × 0.085; AAPR: albumin [g/L] / ALP [IU/L]; AGR: albumin [g/L] / globulin [g/L]; GPR: GGT [μ/L] / PLT; HALP: HGB × albumin [g/L] × LY / PLT; MLR: MO / LY; NLR: NE / LY; PLR: PLT / LY; PNI: albumin [g/L] + 5 × LY; SII: PLT × NE / LY; TBS: $\sqrt{{maximum\,tumor\,diameter\,\left(cm\right)}^{2}+{tumor\,number}^{2}}$; TTV: 4/3 × 3.14 × maximum tumor radius [cm]^3^; Additionally, the optimal cut-off values for each index were calculated using X-tile software.

### Follow-up

Patient follow-up in this study commenced after the surgical procedure. Follow-up evaluations were conducted monthly for the first year post-surgery, followed by assessments every three months for the subsequent two years. The final follow-up was conducted until January 2023. Overall survival (OS) was defined as the duration from the date of radical surgical resection to the last follow-up or death from any cause. Recurrence-free survival (RFS) was defined as the duration from the date of radical surgical resection to the last follow-up, or until tumor progression or death from any cause occurred.

### Construction of TIIN score and evaluation of prognostic value

The variables derived from the 16 indicators were subjected to screening using the Least Absolute Shrinkage and Selection Operator (LASSO) regression model. Subsequently, the TIIN score was calculated based on the variable coefficients obtained from the LASSO regression. The optimal cutoff value for the TIIN score was determined using the X-tile software (Yale University, New Haven, CT, USA). Furthermore, the association between the TIIN score and clinical and pathological indicators was examined using the chi-square test. Additionally, the predictive capability of the TIIN score was assessed by constructing receiver operating characteristic (ROC) curves for the training set and validation set, considering a follow-up period of 1–3 years.

### Development and assessment of nomogram

The independent risk factors for OS and RFS in ICC were identified using univariate Cox regression analysis and multivariate inverse stepwise Cox regression modeling. Subsequently, prediction models for OS and RFS were constructed using nomograms. The accuracy of the models was evaluated by plotting ROC survival curves, calibration curves and decision curve analysis (DCA) based on both the training and validation sets.

### Statistical analysis

In our study, the normality of continuous variables was assessed using the Kolmogorov–Smirnov test. Normally distributed variables were presented as mean ± standard deviation (SD), and non-normally distributed variables were reported as median (interquartile range, IQR). Statistical comparisons between groups were conducted using the Student’s t-test for normally distributed variables and the Mann–Whitney rank sum test for non-normally distributed variables. Categorical variables were compared using either the chi-square test or Fisher’s exact test to analyze patients’ baseline characteristics. Univariate analysis was conducted using Cox survival analysis, while multivariate analysis was performed using the Cox inverse stepwise regression model. IBM SPSS software (version 26.0) was used for all statistical analyses. LASSO regression, construction, evaluation of predictive models, and data visualization were performed using R software (version 4.2.1).

## Results

### Patient baseline characteristics

A total of 418 patients who underwent radical surgical resection for ICC were included in this multicenter study. The median age of the patients was 59 years (range: 28–80), with 230 (55.0%) males and 188 (45.0%) females. The median follow-up period was 12 months (range: 1–91), and the 1-year, 2-year, and 3-year OS rates were 52.2%, 24.6%, and 10.5%, respectively. Correlation analysis of clinical baseline indicators and pathological characteristics in the training set (*n* = 272) and validation set (*n* = 146) revealed a balanced distribution between both cohorts (*p* > 0.05, Table 1).

**Table 1:** Comparison of clinicopathological characteristics in training and validation sets

Variables	All patients (*n* = 418)	Training set (*n* = 272)	Validation set (*n* = 146)	*P* value
Sex				0.945
Male	230(55.0%)	150(55.1%)	80(54.8%)	
Female	188(45.0%)	122(44.9%)	62(45.2%)	
Age (years)				0.311
≤ 65	267(63.9%)	169(62.1%)	98(67.1%)	
> 65	151(36.1%)	103(37.9%)	48(32.9%)	
Obstructive jaundice				0.568
No	366(87.6%)	240(88.2%)	126(86.3%)	
Yes	52(12.4%)	32(11.8%)	20(13.7%)	
HBV infection				0.830
No	272(65.1%)	176(64.7%)	96(65.8%)	
Yes	146(34.9%)	96(35.3%)	50(34.2%)	
AFP (ng/ml)				0.929
< 20	357(85.4%)	232(85.3%)	125(85.6%)	
≥ 20	61(14.6%)	40(14.7%)	21(14.4%)	
CEA (ng/ml)				0.472
< 5	271(64.8%)	173(63.6%)	98(67.1%)	
≥ 5	147(35.2%)	99(36.4%)	48(32.9%)	
CA19-9 (U/ml)				0.766
< 37	162(38.8%)	104(38.2%)	58(39.7%)	
≥ 37	256(61.2%)	168(61.8%)	88(60.3%)	
Child–Pugh Grade				0.647
Grade A	374(89.5%)	242(89.0%)	132(90.4%)	
Grade B	44(10.5%)	30(11.0%)	14(9.6%)	
Tumor differentiation				0.973
Well	44(10.5%)	28(10.3%)	16(11.0%)	
Moderate	306(73.2%)	200(73.5%)	106(72.6%)	
Poor	68(10.5%)	44(16.2%)	24(11.0%)	
Perineural invasion				0.309
No	226(54.1%)	152(55.9%)	74(50.7%)	
Yes	192(45.9%)	120(44.1%)	72(49.3%)	
Microvascular invasion				0.580
No	231(55.3%)	153(56.3%)	78(53.4%)	
Yes	187(44.7%)	119(43.7%)	68(46.6%)	
AJCC 8th edition T stage				0.224
T_1_/T_2_	345(82.5%)	220(80.9%)	125(85.6%)	
T_3_/T_4_	73(17.5%)	52(19.1%)	21(14.4%)	
AJCC 8th edition N stage				0.467
N_0_	303(72.5%)	194(71.3%)	109(74.7%)	
N_1_	115(27.5%)	78(28.7%)	37(25.3%)	
AJCC 8th edition M stage				0.435
M_0_	412(98.6%)	269(98.9%)	113(97.9%)	
M_1_	6(1.4%)	3(1.1%)	3(2.1%)	
ALBI	-2.67(-2.73—-2.61)	-2.69(-2.77- -2.63)	-2.61(-2.72- -2.51)	0.194
ALT (ng/ml)	50(43–58)	50(40–59)	51(38–65)	0.852
AST (ng/ml)	46(40–52)	47(39–55)	45(36–54)	0.781
AAPR	0.38(0.36–0.40)	0.39(0.36–0.41)	0.37(0.34–0.41)	0.545
AGR	1.48(1.45–1.52)	1.49(1.45–1.53)	1.47(1.41–1.53)	0.664
FIB	3.51(3.40–3.62)	3.46(3.32–3.61)	3.59(3.41–3.78)	0.282
GPR	1.09(0.87–1.30)	1.04(0.80–1.27)	1.18(0.76–1.59)	0.530
HALP	48.0(39.4–56.5)	51.3(38.3–64.4)	41.7(38.0–45.4)	0.292
MLR	0.35(0.32–0.39)	0.36(0.31–0.40)	0.35(0.29–0.40)	0.835
NLR	3.63(3.30–3.96)	3.57(3.17–3.96)	3.75(3.13–4.36)	0.611
PLR	155(144–166)	157(141–173)	152(140–160)	0.681
PNI	48.8(47.6–50.0)	49.4(47.7–51.1)	47.8(46.6–49.0)	0.210
PT	12.3(12.2–12.4)	12.3(12.1–12.4)	12.4(12.1–12.6)	0.472
SII	787(704–870)	780(675–885)	800(663–936)	0.830
TBS	6.83(6.48–7.18)	6.84(6.40–7.27)	6.83(6.23–7.43)	0.470
TTV	323(262—385)	319(244–395)	331(221–440)	0.207

*AFP* Alpha fetoprotein, *CEA* Carcinoembryonic antigen, *CA19-9* Carbohydrate antigen199, *AJCC 8th edition* the 8th edition of the American Joint Committee on Cancer, *AAPR* Albumin–alkaline phosphatase ratio, *AGR* Albumin–globulin ratio; albumin–bilirubin (ALBI), *ALT* Alanine aminotransferase, *AST* Aspartate aminotransferase, *FIB* Fibrinogen, *GPR* Gamma-glutamyl transpeptidase-to-platelet ratio, *HALP* Hemoglobin-albumin-lymphocytes-platelets, *MLR* Monocyte-to-lymphocyte ratio, *NLR* Neutrophil-to-lymphocyte ratio, *PLR* Platelet-to-lymphocyte ratio, *PNI* Prognostic nutritional index, *PT* Prothrombin time, *SII* Systemic immune inflammation index, *TBS* Tumor burden score, *TTV* Total tumor volume

### Survival analysis of TIIN score and other indicators

**Fig. 1:**
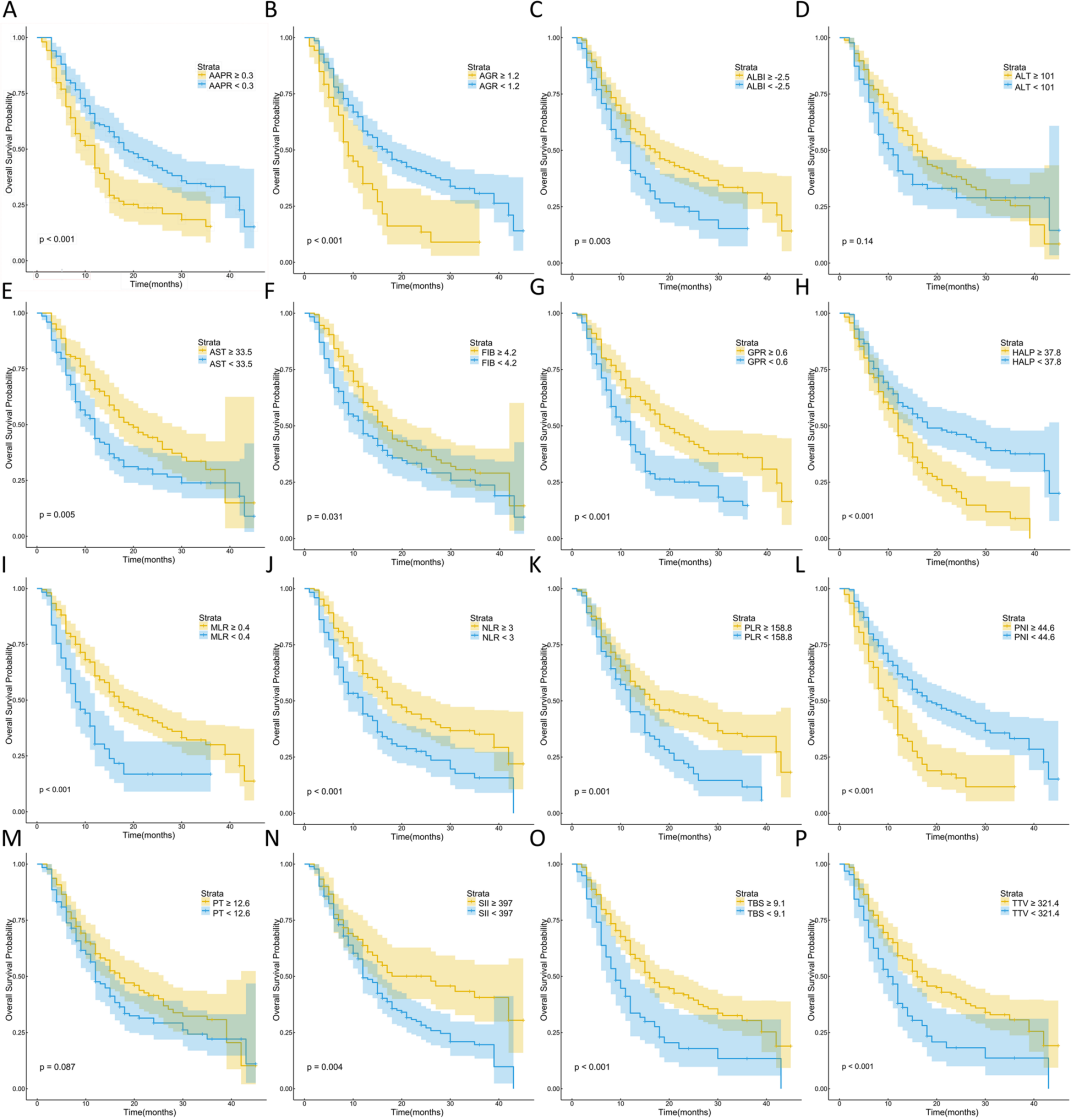
Kaplan–Meier curves for overall survival (OS), stratified by **A** AAPR, **B** AGR, **C** ALBI, **D** ALT, **E** AST, **F** FIB, **G** GPR, **H** HALP, **I** MLR, **J** NLR, **K** PLR, **L** PNI, **M** PT, **N** SII, **O** TBS and **P** TTV in patients with ICC. ICC, intrahepatic cholangiocarcinoma; AAPR, albumin–alkaline phosphatase ratio; AGR, albumin–globulin ratio; albumin–bilirubin (ALBI); ALT, alanine aminotransferase; AST, aspartate aminotransferase; FIB, fibrinogen; GPR, gamma-glutamyl transpeptidase-to-platelet ratio; HALP, hemoglobin-albumin-lymphocytes-platelets; MLR, monocyte-to-lymphocyte ratio; NLR, neutrophil-to-lymphocyte ratio; PLR, platelet-to-lymphocyte ratio; PNI, prognostic nutritional index; PT, prothrombin time; SII, systemic immune inflammation index; TBS, tumor burden score; TTV, total tumor volume

**Fig. 2:**
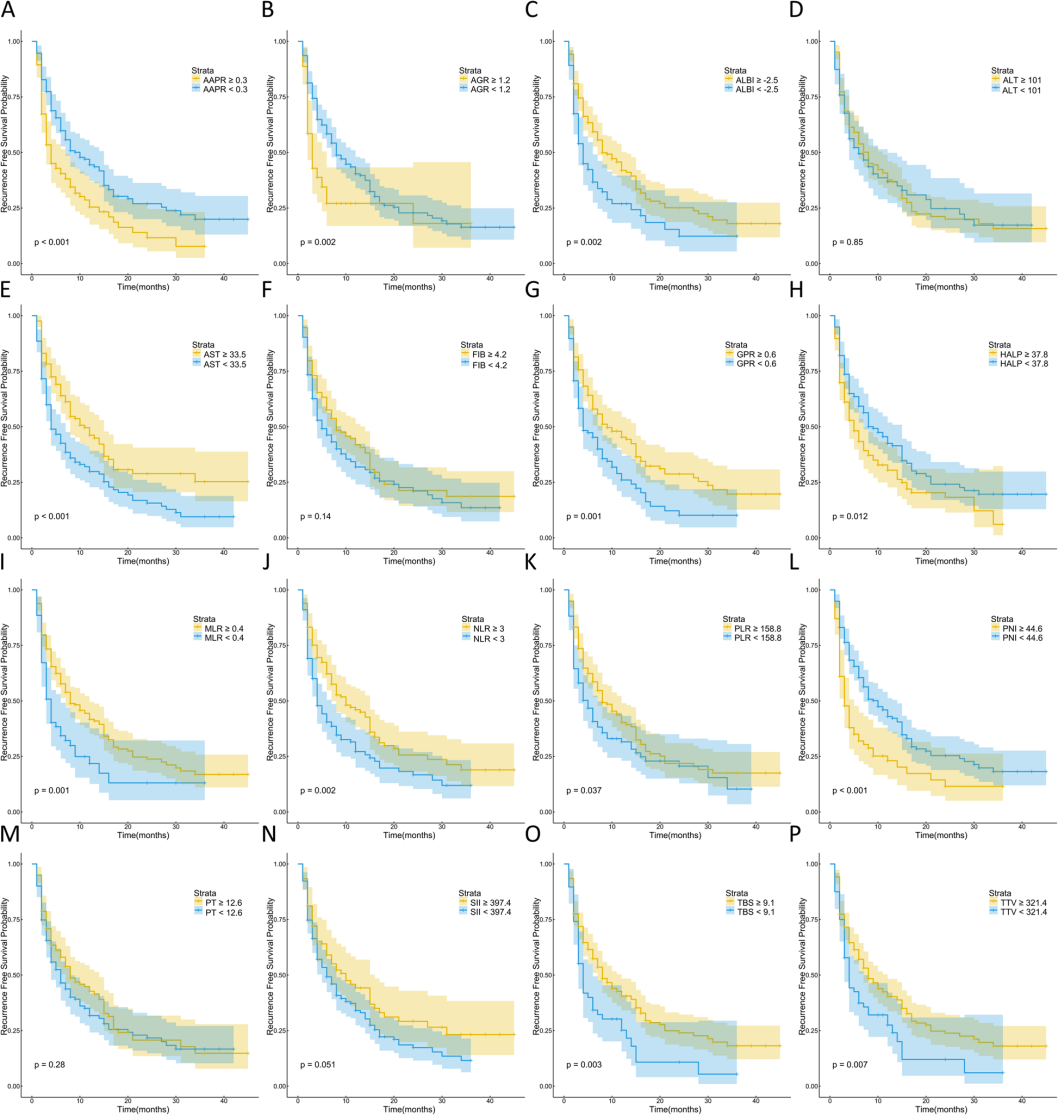
Kaplan–Meier curves for recurrence-free survival (RFS), stratified by **A** AAPR, **B** AGR, **C** ALBI, **D** ALT, **E** AST, **F** FIB, **G** GPR, **H** HALP, **I** MLR, **J** NLR, **K** PLR, **L** PNI, **M** PT, **N** SII, **O** TBS and **P** TTV in patients with ICC

**Fig. 3:**
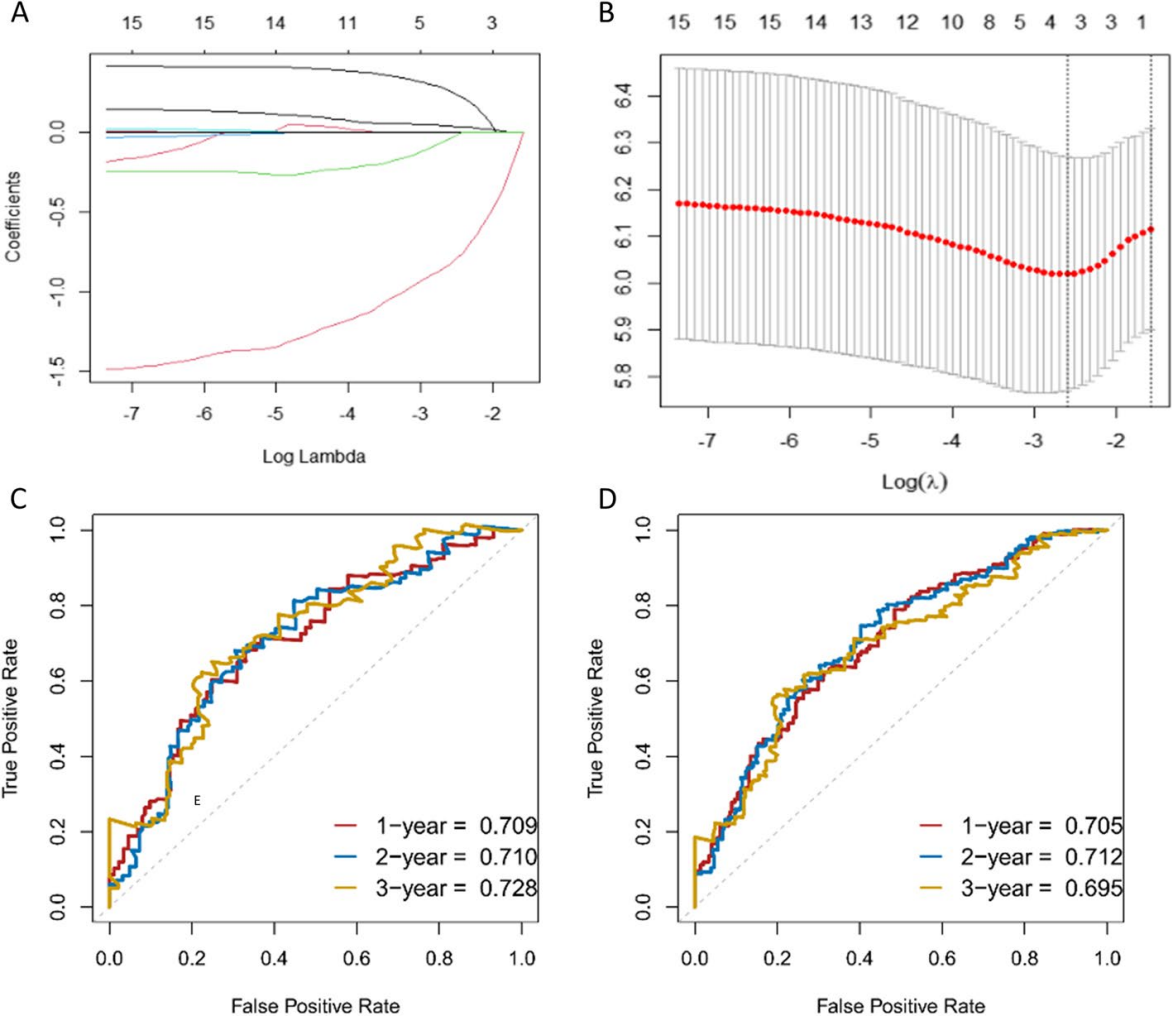
Construction of the TIIN score using the LASSO Cox regression model. **A** Partial likelihood deviance for LASSO coefficient profiles. The red dots represent the partial likelihood values, the grey lines represent the standard error (SE), and the vertical dotted line shows the optimal values by 1-s.e. **B** Least absolute shrinkage and selection operator (LASSO) coefficient profiles of 16 tumor morphology, immune function, inflammatory levels, and nutritional status related biomarkers. The ROC curves for predicting OS at 1-, 2-, and 3-years in the training set (**C**) and the validation set (**D**). ROC, receiver operating characteristic

**Fig. 4:**
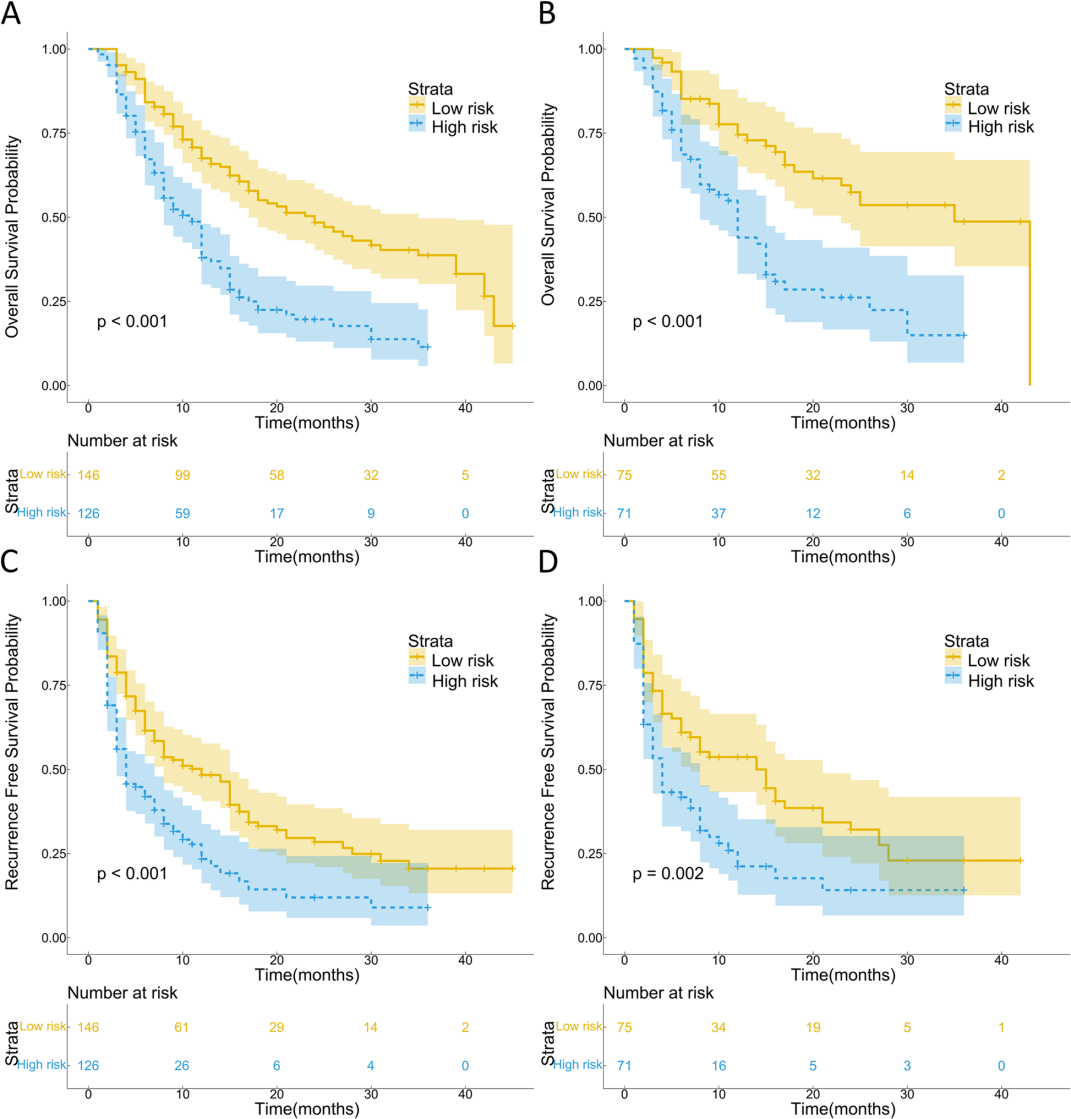
Prognostic implications of the TIIN score. Kaplan–Meier curves of OS (**A**) and RFS (**B**) for patients in the low- and high-risk groups according to the TIIN score in the training set. Kaplan–Meier curves of OS (**C**) and RFS (**D**) for patients in the low- and high-risk groups according to the TIIN score in the validation set

Kaplan–Meier survival analysis demonstrated significant correlations between 14 out of the 16 indicators (excluding ALT and PT) and both OS and RFS (Figs. 1 and 2). Subsequently, we conducted LASSO regression analysis on the 16 indicators, revealing that AAPR, AGR, MLR, and TBS were significantly associated with prognosis (Fig. 3A and B). Utilizing the coefficients derived from the LASSO regression analysis, we constructed the TIIN score as follows: Risk score = 0.0407*TBS + 0.2619*MLR—0.8255*AAPR—0.0409*AGR. We categorized the TIIN score into Low risk (score > 0) and High risk (score ≤ 0) groups. We further examined the correlation between the TIIN score and clinical and pathological features in the training and validation sets. The results demonstrated a significant association between the TIIN score and obstructive jaundice, CEA, CA19-9, Child–pugh grade, perineural invasion, and the 8th edition of the AJCC N stage, which further proved that the TIIN score had a good correlation with clinical and pathological features (Table 2). Comparative analysis of the TIIN score and the area under the curve (AUC) of the 16 individual indicators revealed a significantly higher predictive value for prognosis with the TIIN score (Supplementary Figure 1). Additionally, ROC curves were plotted for 1–3 year OS using the TIIN score in both the training and validation sets, yielding AUC values of 0.705, 0.712, and 0.695 for the training set, and 0.709, 0.710, and 0.728 for the validation set, respectively (Fig. 3C and D). Kaplan–Meier survival curves demonstrated a significant association between the Low risk group based on the TIIN score and better OS and RFS (Fig. 4A-D). Furthermore, univariate and multivariate reverse stepwise Cox regression analyses were performed on the TIIN score, along with other clinical and pathological characteristics in the training set. The results indicated that the TIIN score was an independent risk factor for both OS (hazard ratio [HR] = 1.815, 95%CI = 1.315–2.505, *p* < 0.001) and RFS (HR = 1.540, 95%CI = 1.138–2.083, *p* < 0.001) (Table 3). Overall, the TIIN score exhibited excellent predictive ability as a composite indicator reflecting tumor morphology, immunity, inflammatory level, and nutritional status.

**Table 2:** Relationship of the TIIN score with clinicopathological characteristics of intrahepatic cholangiocarcinoma (ICC) after radical resection in the training and validation sets

	Training set				Validation set			
	Low risk	High risk	X^2^	*p*-value	Low risk	High risk	X^2^	*p*-value
Sex			0.132	0.716			0.001	0.975
Male	82(56.2%)	68(54.0%)			41(54.7%)	39(54.9%)		
Female	64(43.8%)	58(46.0%)			34(45.3%)	32(45.1%)		
Age			0.104	0.747			0.341	0.559
≤ 65	92(63.0%)	77(61.1%)			52(69.3%)	46(64.8%)		
> 65	54(37.0%)	49(38.9%)			23(30.7%)	25(35.2%)		
Obstructive jaundice			9.523	0.002			6.451	0.011
No	137(93.8%)	103(81.7%)			70(93.3%)	56(78.9%)		
Yes	9(6.2%)	23(18.3%)			5(6.7%)	15(21.1%)		
HBV infection			8.519	0.004			3.440	0.064
No	83(56.8%)	93(73.8%)			44(58.7%)	52(73.2%)		
Yes	63(43.2%)	33(26.2%)			31(41.3%))	19(26.8%)		
AFP (ng/ml)			1.468	0.226			0.010	0.920
< 20	121(82.9%)	111(88.1%)			64(85.3%)	61(85.9%)		
≥ 20	25(17.1%)	15(11.9%)			11(14.7%)	10(14.1%)		
CEA (ng/ml)			14.640	< 0.001			9.312	0.002
< 5	108(74.0%)	65(51.6%)			59(78.7%)	39(54.9%)		
≥ 5	38(26.0%)	61(48.4%)			16(21.3%)	32(45.1%)		
CA19-9 (U/ml)			12.583	< 0.001			7.710	0.005
< 37	70(47.9%)	34(27.0%)			38(50.7%)	20(28.2%)		
≥ 37	76(52.1%)	92(73.0%)			37(49.3%)	51(71.8%)		
Child–Pugh grade			7.602	0.006			5.557	0.018
Grade A	137(93.8%)	105(83.3%)			72(96.0%)	60(84.5%)		
Grade B	9(6.2%)	21(16.7%)			3(4.0%)	11(15.5%)		
Tumor differentiation			3.441	0.064			3.740	0.053
Well / Moderate	128(87.7%)	100(79.4%)			67(89.3%)	55(77.5%)		
Poor	18(12.3%)	26(20.6%)			8(10.7%)	16(22.5%)		
Perineural invasion			7.810	0.005			8.858	0.003
No	93(63.7%)	59(46.8%)			47(62.7%)	27(38.0%)		
Yes	53(36.3%)	67(53.2%)			28(37.3%)	44(62.0%)		
Microvascular invasion			7.106	0.008			2.680	0.102
No	93(63.7%)	60(47.6%)			45(60.0%)	33(46.5%)		
Yes	53(36.3%)	66(52.4%)			30(40.0%)	38(53.5%)		
AJCC 8th edition T stage			0.349	0.554			0.138	0.710
T_1_/T_2_	120(82.2%)	100(79.4%)			65(86.7%)	60(84.5%)		
T_3_/T_4_	26(17.8%)	26(20.6%)			10(13.3%)	11(15.5%)		
AJCC 8th edition N stage			8.538	0.003			5.229	0.022
N_0_	115(78.8%)	79(62.7%)			62(82.7%)	47(66.2%)		
N_1_	31(21.2%)	47(37.3%)			13(17.3%)	24(33.8%)		
AJCC 8th edition M stage			0.505	0.477			0.399	0.528
M_0_	145(99.3%)	124(98.4%)			74(98.7%)	69(97.2%)		
M_1_	1(0.7%)	2(1.6%)			1(1.3%)	2(2.8%)

**Table 3:** Univariate and multivariate analyses of the prognosis for intrahepatic cholangiocarcinoma (ICC) after radical resection in the training set

	OS				RFS			
	Univariate analysis		Multivariate analysis		Univariate analysis		Multivariate analysis	
	HR (95%CI)	*p*-value	HR (95%CI)	*p*-value	HR (95%CI)	*p*-value	HR (95%CI)	*p*-value
TIIN score								
High risk vs. Low risk	2.314(1.695–3.157)	< 0.001	1.815(1.315–2.505)	< 0.001	1.799(1.341–2.414)	< 0.001	1.540(1.138–2.083)	0.005
Sex								
Female vs. Male	0.917(0.678–1.240)	0.573			1.020(0.764–1.362)	0.893		
Age (years)								
> 65 vs. ≤ 65	1.012(0.741–1.381)	0.942			0.815(0.604–1.101)	0.182		
Obstructive jaundice								
Yes vs. no	1.062(0.826–1.365)	0.640			0.995(0.793–1.249)	0.968		
HBV infection								
Yes vs. no	0.735(0.532–1.014)	0.061			0.866(0.639–1.173)	0.352		
AFP (ng/ml)								
≥ 20 vs. < 20	0.857(0.556–1.322)	0.486			1.070(0.720–1.591)	0.738		
CEA (ng/ml)								
≥ 5 vs. < 5	1.912(1.409–2.595)	< 0.001	1.450(1.047–2.008)	0.025	1.453(1.080–1.953)	0.013		
CA19-9 (U/ml)								
≥ 37 vs. < 37	2.054(1.471–2.869)	< 0.001	1.524(1.067–2.177)	0.021	1.829(1.343–2.490)	< 0.001	1.716(1.247–2.361)	0.001
Child–Pugh Grade								
Grade A vs. Grade B	1.650(1.053–2.585)	0.029			1.108(0.681–1.803)	0.678		
Tumor differentiation								
Poor vs. Moderate/well	2.064(1.413–3.013)	< 0.001	2.111(1.427–3.122)	< 0.001	1.081(1.248–2.598)	0.002	1.772(1.216–2.582)	0.003
Perineural invasion								
Yes vs. no	1.589(1.169–2.159)	0.003			1.245(0.933–1.663)	0.136		
Microvascular invasion								
Yes vs. no	1.911(1.407–2.597)	< 0.001	1.482(1.074–2.044)	0.017	1.496(1.118–2.002)	0.007	1.337(1.004–1.805)	0.047
AJCC 8th edition T stage								
T_3_/T_4_ vs. T_1_/T_2_	1.326(0.913–1.925)	0.139			1.177(0.808–1.715)	0.396		
AJCC 8th edition N stage								
N_1_ vs. N_0_	1.897(1.370–2.628)	< 0.001	1.440(1.020–2.031)	0.038	1.476(1.083–2.012)	0.014		
AJCC 8th edition M stage								
M_1_ vs. M_0_	1.595(0.394–6.446)	0.513			0.998(0.247–4.031)	0.998		

### Construction and evaluation of nomograms

**Fig. 5:**
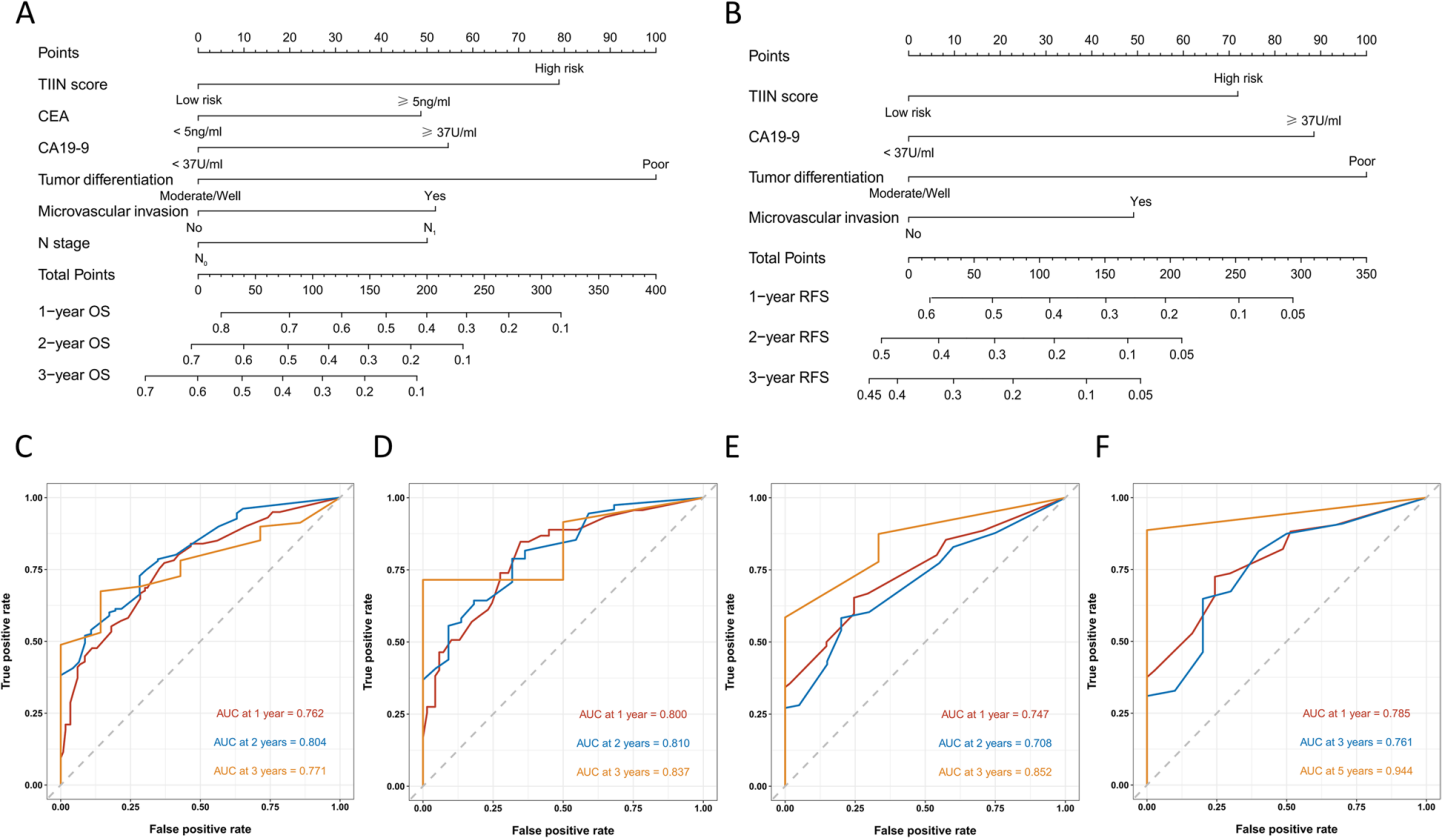
Construction and validation of the nomograms. Nomograms incorporating the TIIN score and other clinicopathological parameters for OS (**A**) and RFS (**B**) prediction in the training set. ROC survival curves of the training set for OS (**C**) and RFS (**D**) based on the model. ROC survival curves of the validation set for OS (**E**) and RFS (**F**) based on the model

**Fig. 6:**
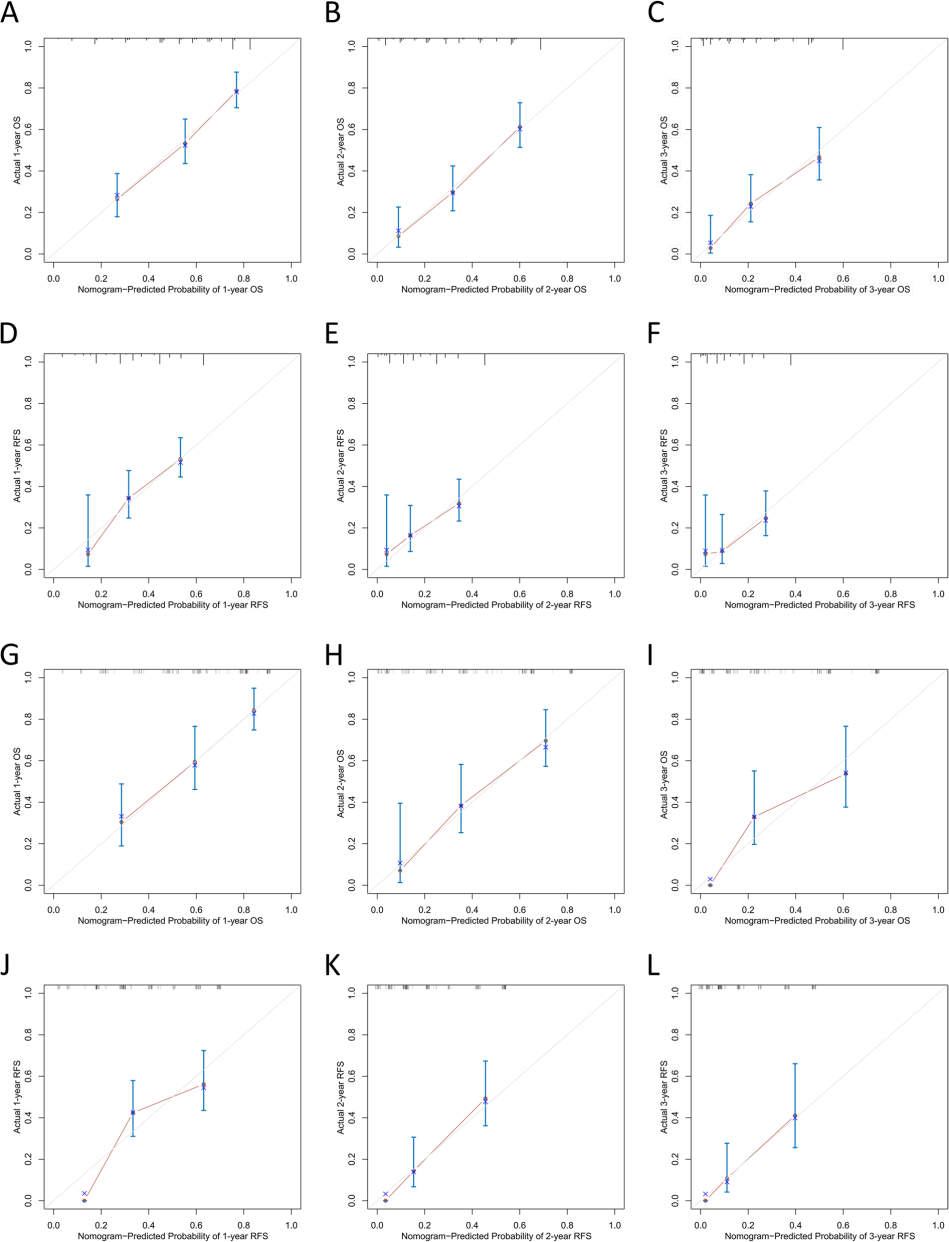
The calibration curves of the nomograms between predicted and observed 1-, 2-, and 3-year OS of patients in the training set (**A**–**C**) and the validation set (**D**–**F**). The calibration curves of the nomograms between predicted and observed 1-, 2-, and 3-year RFS of patients in the training set (**G**–**I**) and the validation set (**J**–**L**) The dashed line of 45° represents the perfect prediction of the nomogram

**Fig. 7:**
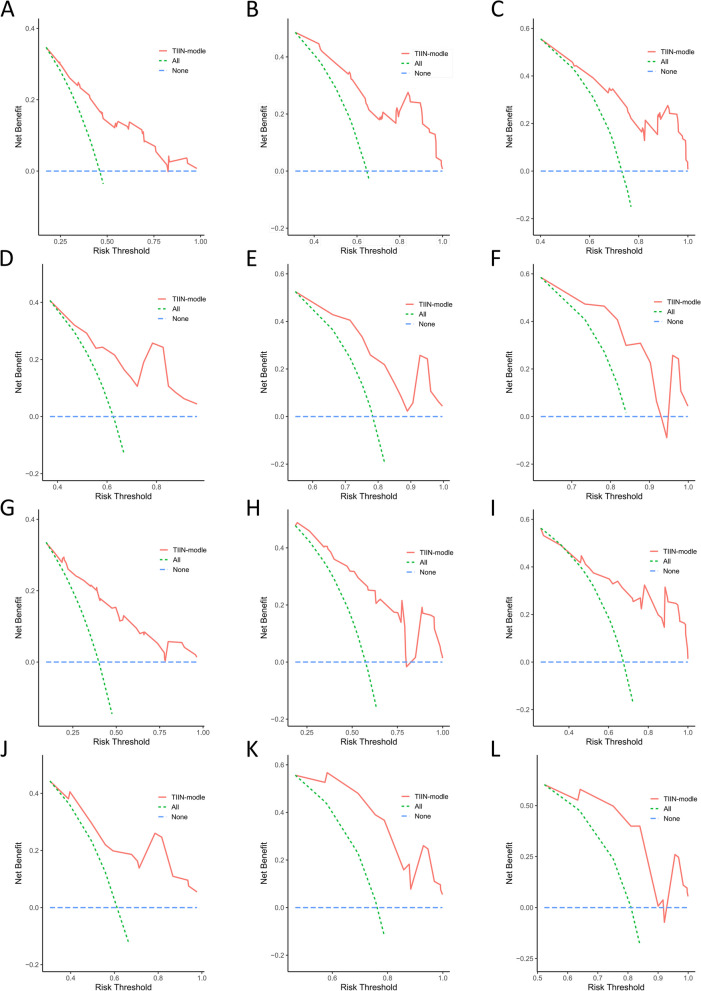
Decision curve analysis (DCA) of OS and RFS prediction by the nomograms. The DCA of the nomogram for 1-year OS (**A**), 2-year OS (**B**), and 3-year OS (**C**) and for 1-year RFS (**D**), 2-year RFS (**E**), and 3-year RFS (**F**) in the training set. DCA of the nomogram for 1-year OS (**G**), 2-year OS (**H**), and 3-year OS (**I**) and for 1-year RFS (**J**), 2-year RFS (**K**), and 3-year RFS (**L**) in the validation set

Multivariate reverse stepwise Cox regression analysis for OS and RFS identified the TIIN score, CEA, CA19-9, tumor differentiation, microvascular invasion, and AJCC 8th edition N stage as predictors for the OS prediction model (Fig. 5A). Similarly, the RFS prediction model included the TIIN score, CA19-9, tumor differentiation, and microvascular invasion (Fig. 5B). ROC survival curves were plotted for the training and validation sets using the predictive models. The AUCs for 1–3 year OS in the training set were 0.762, 0.804, and 0.771, while for the validation set, the AUCs were 0.800, 0.810, and 0.837 (Fig. 5C and D). The AUCs for 1–3 year RFS in the training set were 0.747, 0.708, and 0.852, and for the validation set, the AUCs were 0.785, 0.761, and 0.944 (Fig. 5E and F). Additionally, calibration curves and DCA were plotted for 1–3 years based on the training and validation sets, demonstrating excellent predictive ability of the models for postoperative survival in ICC patients (Figs. 6 and 7).

## Discussion

Radical surgical resection remains the established standard treatment for ICC worldwide [6, 23, 24]. However, the potential benefits of surgery depend on individualized patient characteristics [25]. Numerous studies have demonstrated the influence of tumor morphology, immune function, inflammatory levels, and nutritional status on the prognosis of ICC through various pathways. Recent research has specifically highlighted the significance of personalized indicators such as TTV and TBS in predicting patient outcomes [8, 26]. Additionally, indicators reflecting immune function, inflammatory levels, and nutritional status, such as ALBI, SII, and PNI, have been established as reliable prognostic assessment markers for ICC patients in relevant studies [12, 14, 27, 28].

Integrating multiple evaluation indexes is essential to capture the individualized characteristics of patients, as a single preoperative evaluation index may not provide a comprehensive assessment. In our study, we collected imaging and laboratory test data, evaluating 16 relevant indexes related to tumor morphology, immune function, inflammatory levels, and nutritional status. Our findings demonstrated significant correlations between ALBI, AST, AAPR, AGR, FIB, GPR, HALP, MLR, NLR, PLR, PNI, PT, SII, TBS, TTV, and the prognostic status of patients. Notably, TIIN emerged as the first comprehensive indicator to integrate tumor morphology, immune function, inflammatory levels, and nutritional status. Furthermore, our study revealed that the TIIN score effectively stratifies the prognosis of ICC patients who underwent radical surgical resection. Additionally, the column-line graph prediction model, incorporating the TIIN score and other clinical characteristics, demonstrated excellent predictive accuracy for patient prognosis.

Based on LASSO regression analysis, the TIIN score was constructed using four indicators: TBS, MLR, AAPR, and AGR. This score provides a comprehensive evaluation of tumor morphology, immune function, inflammatory levels, and nutritional status. Previous studies have consistently demonstrated the significant association of these four markers with the prognosis of ICC. TBS, which combines tumor size and number, offers a personalized assessment of tumor characteristics. It has been successfully applied to predict the prognosis of colorectal cancer liver metastases and has shown prognostic value in various cancer types, including HCC and ICC [29–32]. MLR, a representative marker of immune status, reflects the ratio of lymphocytes to monocytes. Elevated MLR has been consistently associated with poorer prognosis in primary liver malignancies, as indicated by several studies [33–35]. AAPR, calculated from albumin and alkaline phosphatase, and AGR, calculated from albumin and globulin, reflect both inflammatory and nutritional status. In our study, we also found that lower levels of AAPR and AGR were often associated with poorer prognosis, which is consistent with the previous findings [36–39]. Therefore, the TIIN score, incorporating TBS, MLR, AAPR, and AGR, provides a comprehensive and validated prognostic tool for ICC patients.

The aforementioned indicators can all be derived from preoperative imaging and laboratory results, allowing for the simple calculation of the TIIN score as an integrated indicator. By incorporating these metrics, the TIIN score provides a comprehensive assessment of individualized patient characteristics, encompassing tumor characteristics, immune function, inflammatory levels, and nutritional status. Our multicenter study further confirmed that higher TIIN scores were significantly associated with improved OS and RFS. Furthermore, the column-line graph prediction model, based on the TIIN score and multivariate Cox regression results, exhibited favorable predictive capability, as evidenced by the assessment of its ROC curve, calibration curve and DCA.

Our study has certain limitations that should be acknowledged. Firstly, although it was a multicenter retrospective study, the sample size was limited to 418 cases, and all the participants were from China. Thus, validation from other centers worldwide is necessary to establish broader applicability. Secondly, being a retrospective study, it is susceptible to selective bias, particularly since we only included patients who underwent surgical resection without prior treatments. Lastly, despite our efforts to minimize confounding factors, the influence of individual variations on the test indicators cannot be entirely eliminated. While we have made attempts to mitigate the impact of confounding factors, individual differences may still have an effect on each test index.

## Conclusion

In our multicenter analysis of 418 patients, we observed a significant association between the TIIN score, an integrated indicator encompassing tumor morphology, immunity, inflammation level, and nutritional status, and the prognosis of ICC patients. Additionally, the nomogram prediction model, incorporating the TIIN score and other clinical indicators, demonstrated strong predictive capability. These findings offer valuable insights for the development of individualized treatment plans for ICC patients in the future.

### Supplementary Information

Supplementary Material 1.

Supplementary Material 2.

## Data Availability

The datasets used and analysed during the current study available from the corresponding author on reasonable request.

## Abbreviations

ICC: Intrahepatic cholangiocarcinoma
HCC: Hepatocellular carcinoma
TIIN score: Tumor morphology immune inflammatory nutritional score
AJCC: The American Joint Committee on Cancer
HBV: Hepatitis B virus
AFP: Alpha fetoprotein
CEA: Carcinoembryonic antigen
CA19-9: Carbohydrate antigen199
ALT: Alanine transaminase
AST: Aspartate transaminase
ALP: Alkaline phosphatase
GGT: Gamma-glutamyltransferase
PT: Prothrombin time
FIB: Fibrinogen
WBC: White blood cell count
HGB: Hemoglobin
LY: Lymphocyte count
NE: Neutrophil count
MO: Monocyte count
PLT: Platelet count
ALBI: Albumin–bilirubin
AAPR: Albumin-alkaline phosphatase ratio
AGR: Albumin–globulin ratio
GPR: Gamma-glutamyl transpeptidase-to-platelet ratio
HALP: Hemoglobin-albumin-lymphocytes-platelets
MLR: Monocyte-to-lymphocyte ratio
NLR: Neutrophil-to-lymphocyte ratio
PLR: Platelet-to-lymphocyte ratio
PNI: Prognostic nutritional index
SII: Systemic immune inflammation index
TBS: Tumor burden score
TTV: Total tumor volume
OS: Overall survival
RFS: Recurrence-free survival
LASSO: The Least Absolute Shrinkage and Selection Operator
ROC: Receiver operating characteristic
DCA: Decision curve analysis
SD: Standard deviation
IQR: Interquartile range
