# Supplementary material for: Construction and validation of a novel tumor morphology immune inflammatory nutritional score (TIIN score) for intrahepatic cholangiocarcinoma: a multicenter study
Source: BMC Cancer. 2024 May 23;24:630. doi: 10.1186/s12885-024-12375-7 (PMC11112867; doi:10.1186/s12885-024-12375-7)
Supplement: Supplementary file 1 — Supplementary Material 1. [file 12885_2024_12375_MOESM1_ESM.docx]

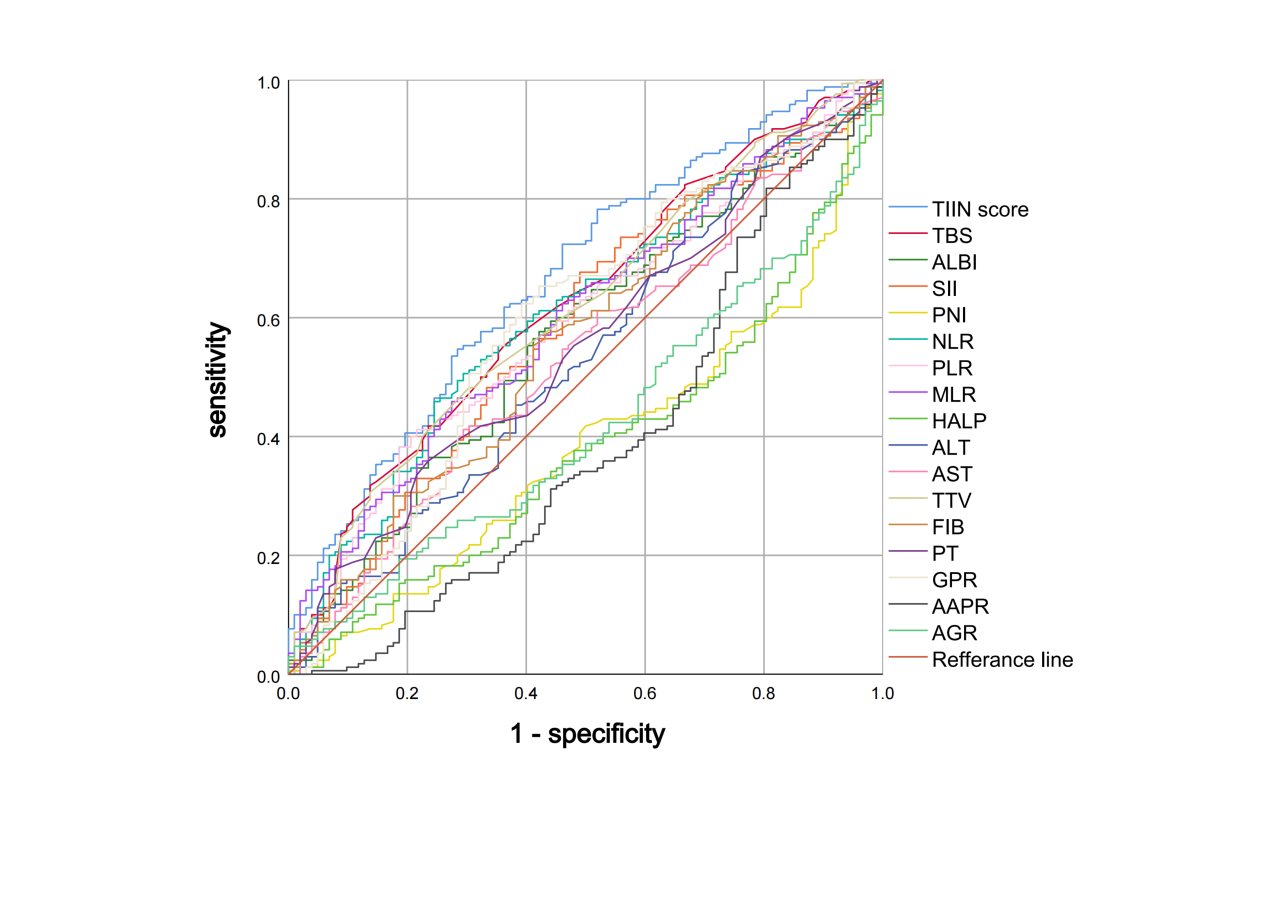


SUPPLEMENTARY FIGURE 1

ROC curves showing the predictive power of the TIIN score compared to a single indicator in the training set. TIIN score, tumor morphology immune inflammatory nutritional score; AAPR, albumin–alkaline phosphatase ratio; AGR, albumin–globulin ratio; albumin–bilirubin (ALBI); ALT, alanine aminotransferase; AST, aspartate aminotransferase; FIB, fibrinogen; GPR, gamma-glutamyl transpeptidase-to-platelet ratio; HALP, hemoglobin-albumin-lymphocytes-platelets; MLR, monocyte-to-lymphocyte ratio; NLR, neutrophil-to-lymphocyte ratio; PLR, platelet-to-lymphocyte ratio; PNI, prognostic nutritional index; PT, prothrombin time; SII, systemic immune inflammation index; TBS, tumor burden score; TTV, total tumor volume.
