# Supplementary material for: Construction and validation of a novel tumor morphology immune inflammatory nutritional score (TIIN score) for intrahepatic cholangiocarcinoma: a multicenter study
Source: BMC Cancer. 2024 May 23;24:630. doi: 10.1186/s12885-024-12375-7 (PMC11112867; doi:10.1186/s12885-024-12375-7)
Supplement: Supplementary file 2 — Supplementary Material 2. [file 12885_2024_12375_MOESM2_ESM.docx]

**Supplementary CT information**

**Scanning parameters for CT abdomen include:**

tube voltage of 120 kV, automatic tube current, rotation time of 0.4, 0.5 or 0.75 s; detector collimation of 64 x 0.625 mm, 128 x 0.625 mm or 16 x 0.625 mm, field of view of (300-500) mm x (300-500) mm, matrix of 512 x 512, layer spacing of 5 mm, layer thickness of 5 mm.

After CT scanning of the abdomen, an enhanced CT scan of the abdomen was performed, starting 25-30 seconds after arterial phase contrast injection and 60-70 seconds after intravenous phase contrast injection, with intravenous injection of contrast agent (ultravist 370, Bayer Schering Pharma, Berlin, Germany) at a dose of 1.5 ml/kg and an injection rate of 3.0 to 3.5 ml/s using a high-pressure injector Ulrich CT Plus 150 (Ulrich Medical, Ulm, Germany).
